# Retromer-dependent lysosomal stress in Parkinson's disease

**DOI:** 10.1098/rstb.2022.0376

**Published:** 2024-04-08

**Authors:** Dario R. Alessi, Peter J. Cullen, Mark Cookson, Kalpana M. Merchant, Scott A. Small

**Affiliations:** ^1^ MRC Protein Phosphorylation and Ubiquitylation Unit, School of Life Sciences, University of Dundee, Dow Street, Dundee DD1 5EH, UK; ^2^ School of Biochemistry, University of Bristol, Biomedical Sciences Building, Bristol BS8 1TD, UK; ^3^ Laboratory of Neurogenetics, National Institute on Aging, National Institutes of Health, Bethesda, MD 20892, USA; ^4^ Department of Neurology, Feinberg School of Medicine, Northwestern University, Chicago, IL 60208, USA; ^5^ Department of Neurology and the Taub Institute for Research on Alzheimer’s Disease and the Aging Brain, Columbia University, New York, NY 10032, USA

**Keywords:** Parkinson's disease, retromer, LRRK2, VPS35

## Abstract

While causative mutations in complex disorders are rare, they can be used to extract a biological pathway whose pathogenicity can generalize to common forms of the disease. Here we begin by relying on the biological consequences of mutations in LRRK2 and VPS35, genetic causes of autosomal-dominant Parkinson's disease, to hypothesize that ‘Retromer-dependent lysosomal stress’ represents a pathway that can generalize to idiopathic Parkinson's disease. Next, we outline a series of studies that can test this hypothesis, including the development of biomarkers of pathway dysfunction. If validated, the hypothesis can suggest a unified mechanism of disease and might inform future diagnostic and therapeutic investigations.

This article is part of a discussion meeting issue ‘Understanding the endo-lysosomal network in neurodegeneration’.

## 1. Introduction

Pathobiologic mechanisms associated with rare causal gene variants have been shown to provide insights into those underlying ‘sporadic’ common forms of the disorder. A canonical example is how biological insight into rare mutations in the gene encoding the Low-Density Lipoprotein Receptor (LDRLR) were used to implicate cholesterol metabolism, a cellular pathway that then generalized to idiopathic hypercholesterolemia and cerebrovascular disease. A more recent example, and one relevant to neurodegenerative diseases and the endo-lysosomal network, is provided by mutations in the Sortilin-Related Receptor 1 (SORL1), which has recently emerged as only the fourth gene that causes a rare, early-onset form of Alzheimer's disease [1,2]. Biological studies have first established that SORL1 triggers the disease by disrupting Retromer-dependent endosomal recycling, and second, have shown that this pathway can generalize to the common sporadic form of the disease (as reviewed in [3]).

Here we rely on the emerging biology of the endo-lysosomal network, on the one hand, and recent insight into rare causal Parkinson's disease (PD) variants on the other to extract a biological pathway that we hypothesize underlies at least a subset of sporadic PD cases. We start by reviewing two key endosomal trafficking pathways that when disrupted differentially ramify throughout the endo-lysosomal network, and how each is differentially regulated by a similar, but not identical, trafficking machine. Next, we review recently described biological consequences of autosomal-dominant PD mutations in Leucine-rich Repeat Kinase 2 (LRRK2) and Vacuolar Protein Sorting 35 (VPS35), explaining how their encoded proteins affect the endo-lysosomal network. Together, we develop a hypothesis that ‘Retromer-dependent lysosomal stress’ might be a pathway that generalizes from monogenic forms to idiopathic PD and outline a series of experiments that are needed to validate the hypothesis.

## 2. Retromer and the endo-lysosomal network

Among its many organelles, the endosome is often considered a central cargo sorting and trafficking station in the eukaryotic cell [4]. The endosome interfaces with the cell surface and the biosynthetic pathway via the trans-Golgi network (TGN). Thus, the endosome acts as a hub for the cell's dominant trafficking itineraries and as a centralized distributer of cargo to cellular sites where they function. For example, receptor-bound proteases are delivered from the TGN to endosomes where the proteases disengage from their receptor. While the proteases remain in the endosomal lumen and are ultimately delivered to the lysosome, the unbound receptors are sorted and exported back to the TGN for future rounds of endosomal delivery. Another key example is provided by the many hundreds of endocytosed cell surface transmembrane proteins that enter the endosomal network, from where they can be trafficked to the TGN and the secretory pathway, the lysosome for degradation, or recycled back to the cell surface to maintain their normal function.

**Figure 1.**
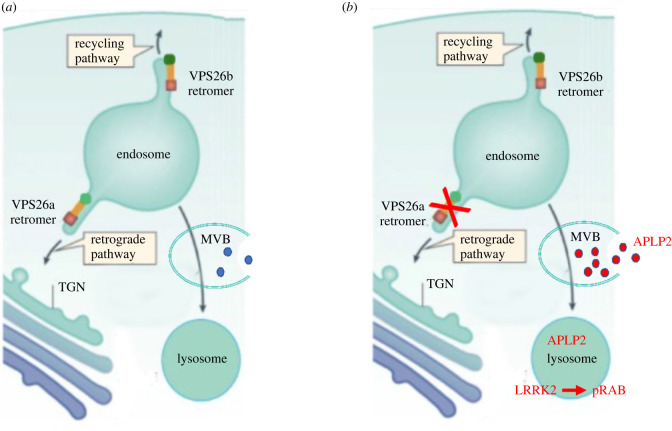
Retromer-dependent lysosomal stress in Parkinson's disease. (*a*) Retromer's retrograde pathway is vital for the forward delivery of proteases to the lysosomal via the endosome. Proteins, such as APLP2, can accumulate in the multivesicular body (MVB) in a Retromer-dependent manner where they can be secreted to the extracellular space. (*b*) PD-associated VPS35 mutations target the retrograde pathway, causing a subtle and specific type of lysosomal stress. Lysosomal stress causes LRRK2 translocation to the lysosomal membranes where it hyperphosphorylates RAB proteins leading to lysosomal dysfunction. Retromer-dependent lysosomal stress should manifest in a lysosomal molecular signature and in a secretion signature.

Similar to how clathrin and its adapter proteins act as a dominant trafficking complex dedicated to the transport of cargo into the endosome from either the TGN or the cell surface, Retromer is a separate multiprotein complex dedicated to the reverse itinerary, the endosomal sorting of cargos back to the TGN, termed retrograde transport, or the trafficking of cargo back to the cell surface, termed the recycling pathway [5,6] (figure 1). Retromer can be considered a multimodular trafficking machine [7], comprised a central core and associated accessory proteins, and studies have been clarifying how it can regulate the two separate transport pathways out of the endosome. For example, Retromer's core module, to which all other accessory proteins connect, is a trimeric complex comprised VPS26-VPS35-VPS29 [5]. VPS26 turns out to have two paralogs, VPS26a and VPS26b. VPS26b can form a distinct heterotrimer in neurons that is preferentially dedicated to the recycling pathway [8] (figure 1). Other Retromer accessory proteins act as adaptors that impose Retromer's specificity to one pathway or the other [9].

As the cell's central trafficking station, impeding endosomal outflow by disrupting Retromer-dependent trafficking will ultimately ramify throughout the cell. Nevertheless, a primary disruption of one Retromer pathway or the other will differentially manifest within the endo-lysosomal network. Disrupting Retromer's recycling pathway will, for example, have a relatively greater effect on cell surface function, and because the pathway recycles hundreds of transmembrane proteins [10], its backlog typically results in endosomal swelling and dysfunction. By contrast, because Retromer's retrograde pathway transports relatively fewer transmembrane proteins back to the TGN, and ultimately is in the service of the lysosome's ability to degrade a vast array of proteins, disrupting this pathway will differentially result in lysosomal dysfunction.

## 3. Autosomal-dominant Parkinson's disease and the endo-lysosomal network

There are variants in three genes that are unequivocally associated with autosomal-dominant PD: the gene encoding alpha-synuclein (SNCA), Leucine-rich Repeat Kinase 2 (LRRK2), and Vacuolar Protein Sorting 35 (VPS35) [11,12]. The endo-lysosomal network has been implicated in alpha-synuclein toxicity because this normally synaptic protein needs to be properly trafficked to the lysosome for degradation. It has been suggested that lysosomal mistrafficking, either of alpha-synuclein itself or of its lysosomal proteases, can accelerate its toxicity by abnormally increasing its accumulation and/or by accelerating lysosomal dysfunction more generally [13]. Therefore, dysfunction of the endo-lysosomal system may modify PD pathogenesis indirectly by effects on alpha-synuclein.

Both *LRRK2* and *VPS35* encode proteins that directly function in endo-lysosomal trafficking. Since it is the backbone of Retromer's core complex, a disease-associated mutation in VPS35 is expected to affect Retromer-dependent trafficking. While this might seem obvious, the PD-associated VPS35 mutation, VPS35[D620N] (a mutation that results in an aspartate to an asparagine substitution at residue 620) turns out to have a relatively subtle molecular consequence by specifically impairing its interactions with FAM21 [14], a key member of the Retromer-related WASH complex [15]. More importantly, studies have shown that the PD-associated VPS35 mutation differentially disrupts Retromer's retrograde pathway, compared to Retromer's recycling pathway, ultimately leading to relatively subtle and specific defects in lysosomal function [16,17].

It is well-established that pathogenic LRRK2 mutations cause kinase hyperactivity [18], and studies suggest that these mutations are associated with lysosomal swelling and dysfunction [19,20] A specific mechanism linking kinase hyperactivity with this cellular phenotype is suggested by molecular screens showing that LRRK2's primary function is to phosphorylate many of the family of Rab proteins [21], which are GTPases that act as effectors in membrane trafficking [22]. Rab8 and Rab10 are the main Rabs hyperphosphorylated by LRRK2 mutations [18], and both Rabs are recruited to damaged lysosomes [23,24].

Several different experimental manipulations that induce lysosomal damage cause a translocation of LRRK2 to lysosomal membranes where it can phenocopy LRRK2 mutations by hyperphosphorylating Rab8 or Rab10 and thereby affect lysosomal structure and function [23–25] Although recruitment and activation of LRRK2 by lysosomal damage is therefore robust, how LRRK2 and phosphorylated Rab proteins are targeted to dysfunctional or stressed lysososmes and the pathways they control at this organelle remain to be further elucidated.

Several studies have suggested a functional link between PD-associated VPS35 and LRRK2 mutations [26–29]. The one study of note that most precisely demonstrated that theVPS35 mutation acts upstream of LRRK2 to regulate its kinase activity and Rab hyperphosphorylation [30] used a range of techniques, different cell lines sourced from animal models and PD patients. Since most studies suggest that they do not directly bind, the precise mechanism for this functional relationship remains unknown. Of note, the VPS35[D620N] mutation enhances LRRK2 pathway activity to a significantly greater extent than PD pathogenic mutations in LRRK2 [30].

## 4. The hypothesis: Retromer-dependent lysosomal stress

While an array of experimental manipulations that induce lysosomal stress can cause LRRK2-mediated Rab hyperphosphorylation and lysosomal dysfunction [31], these manipulations are not physiological. In addition, there are potentially many physiological ways to induce lysosomal stress, and many are not likely to cause PD. It is therefore important to identify specific types of lysosomal stress associated with LRRK2 and VPS35 mutations.

The first assumption of the hypothesis is that Retromer-dependent lysosomal stress exemplifies the type of stress that is specific to PD, and since this leads to translocation of LRRK2 to lysosomal membranes, provides a cellular mechanism by which VPS35 can indirectly regulate LRRK2 activity (figure 1). We hypothesize that a molecular signature typifying PD-associated lysosomal stress can be derived by identifying the lysosomal molecular profile triggered by the PD-associated VPS35 mutation and LRRK2 mutations. While each individual profile might not generalize, the assumption is that the overlap of the two is more likely to reflect the lysosomal stress that might occur in idiopathic PD.

Disrupting Retromer-dependent endosomal trafficking leads to accelerated cellular secretion via ‘unconventional’ endo-lysosomal secretion [32,33]. Specifically, when proteins accumulate in the late-endosome/multivesicular body (MVB), they can be secreted to the extracellular space when the MVB fuses with the cell membrane (figure 1). Proteins can accumulate in the MVB because of increased delivery when Retromer's recycling pathway is disrupted, or because of a protein backlog caused by a decrease in lysosomal degradation when Retromer's retrograde pathway is disrupted.

Accordingly, the second assumption of the hypothesis is that there will be a specific profile of secreted proteins that report on Retromer-dependent lysosomal stress, which can be found in the CSF, blood or even urine. These biomarker(s) can be derived by profiling biofludic alterations induced by the PD-associated VPS35 or LRRK2 mutations. While each individual profile might not generalize, the assumption is that the overlap of the two is more likely to reflect a signature of Retromer-dependent lysosomal stress in idiopathic PD. If this signature is found, investigating other PD-associated genetic mutations might further impose the specificity of the signature to PD. Indeed, a convergence between LRRK2 and VPS35 at the level of a biofluidic biomarker is evident in the recently reported increases in levels of the lysosomal phospholipid, bis(monoacylglycerol)phosphate, in the urine from individuals with pathogenic LRRK2 and VPS35 variants [34].

## 5. Validating the hypothesis and its assumptions

Ideally, the hypothesis is best tested in patients carrying VPS35 or LRRK2 mutations. Nevertheless, mechanistic assumptions undergirding the hypothesis are more readily tested in mouse models harbouring these mutations.

Consistent with the fact that PD is a slowly progressive disorder that typically manifests in midlife or older, these mouse models develop subtle evidence of brain dysfunction in adulthood in the case of LRRK2 mutations [35] or in late-adulthood in the case of the VPS35 mutation [36,37]. Accordingly, in attempting to begin validating the hypothesis, it is important to first determine when and where brain dysfunction is triggered by these mutations. This required first step can be achieved by imaging the mouse models longitudinally using MRI techniques that possess the required microscopic resolution necessary to visualize cortical and subcortical regions, and that are sensitive to either disease-associated functional or structural changes.

Once a specific region or select group of regions are found that overlap in both VPS35 and LRRK2 mouse models these regions can be microdissected. Using lysosomal isolation techniques these ex-vivo samples can be molecularly screened, determining the set of molecules abnormally up or downregulated, and those that overlap in both mouse models. If an overlap is found, this will not only validate the first assumption of the hypothesis, that both mutations converge in inducing a specific ex-vivo signature of Retromer-dependent lysosomal stress, but also will establish a molecular signature of lysosomal stress that we predict will generalize to idiopathic PD.

If a specific region or select group of regions are found to overlap in both VPS35 and LRRK2 mouse models, CSF, blood and urine can be acquired from the mice, thereby testing the hypothesis' second assumption, that there will be a biofluidic biomarker of pathway dysfunction. The assumption can be tested via hypothesis-free mass spectrometry screens, determining an overlap between both mouse models. Alternatively, by relying on prior findings, a hypothesis-driven approach can be taken. For example, previous proteomic screens of the CSF of VPS35 KO mice [38] and a screen of the secreted proteins of VPS35 KO cell lines [32] reliably converged in an increase secretion of APLP2. Since VPS35 depletion will affect Retromer's retrograde pathway, we postulate that APLP2 backlog caused by a decrease in lysosomal degradation leads to its secretion (figure 1). Interestingly, previous studies have implicated APLP2 in PD, showing its involvement in models of PD [39] including LRRK2 mice models [40], and APLP2 is found differentially expressed in nigral regions differentially affected by PD [41] (figure 1*b*).

## 6. Summary

Lysosomal dysfunction has long been implicated in PD's cell biology. The hypothesis that Retromer-dependent lysosomal stress is a specific and potentially unifying pathogenic pathway emerges from recent insight into Retromer biology and its direct and interconnected link to two autosomal-dominant PD mutations.

A key component of testing the hypothesis is that Retromer-dependent lysosomal stress will have a biomarker signature of pathway dysfunction. If validated, these biomarkers will prove most useful for testing whether this pathway is disrupted in idiopathic PD. The biomarker can also be used to test whether Retromer-dependent lysosomal stress occurs in the third autosomal-dominant mutation in SNCA and other PD-risk variants such as GBA1.

Most importantly, if the hypothesis is validated, identified biomarkers for this form of lysosomal dysfunction hold strong promise to accelerate therapeutic development. Unlike other neurodegenerative disorders, such as Alzheimer's disease, PD is notable for its relative dearth of reliable biofluidic biomarkers. If validated, the biomarkers can be used, for example, for highlighting PD patients with lysosomal dysfunction, whether they carry disease-causing mutations or not. Whether testing the current class of LRRK2 kinase inhibitors or testing future drugs targeting the upstream Retromer retrograde pathway, these biomarkers might also be tested for target engagement/target modulation during a clinical trial.

Ultimately, by enabling a precision medicine approach, the biomarkers will improve the success rate of PD therapies by enriching the trial population with subjects most likely to benefit from drugs that target the Retromer-dependent lysosomal stress pathway.

## Data Availability

This article has no additional data.
